# CD1d expression demarcates *CDX4+* hemogenic mesoderm with definitive hematopoietic potential

**DOI:** 10.1016/j.scr.2022.102808

**Published:** 2022-07

**Authors:** J. Philip Creamer, Stephanie A. Luff, Hao Yu, Christopher M. Sturgeon

**Affiliations:** a Department of Medicine, Division of Hematology, Washington University School of Medicine, St. Louis, MO, United States; b Department of Cell, Developmental and Regenerative Biology, Icahn School of Medicine at Mount Sinai, New York, NY, United States; c Black Family Stem Cell Institute, Icahn School of Medicine at Mount Sinai School of Medicine, New York, NY, United States

**Keywords:** Human pluripotent stem cells, Definitive hematopoiesis, Hemogenic mesoderm, Hemogenic endothelium, CD1d, *CDX*

## Abstract

To achieve efficient, reproducible differentiation of human pluripotent stem cells (hPSCs) towards specific hematopoietic cell-types, a comprehensive understanding of the necessary cell signaling and developmental trajectories involved is required. Previous studies have identified the mesodermal progenitors of extra-embryonic-like and intra-embryonic-like hemogenic endothelium (HE), via stage-specific WNT and ACTIVIN/NODAL, with *GYPA/GYPB* (CD235a/b) expression serving as a positive selection marker for mesoderm harboring exclusively extra-embryonic-like hemogenic potential. However, a positive mesodermal cell-surface marker with exclusively intra-embryonic-like hemogenic potential has not been identified. Recently, we reported that early mesodermal expression of *CDX4* critically regulates definitive HE specification, suggesting that CDX4 may act in a cell-autonomous manner during hematopoietic development. To identify *CDX4+* mesoderm, we performed single cell (sc)RNAseq on hPSC-derived mesodermal cultures, revealing *CDX4^hi^* expressing mesodermal populations were uniquely enriched in the non-classical MHC-Class-1 receptor *CD1D.* Flow cytometry demonstrated approximately 60% of KDR+CD34-CD235a- mesoderm was CD1d+, and *CDX4* was robustly enriched within CD1d+ mesoderm. Critically, only CD1d+ mesoderm harbored CD34+ *HOXA+* HE with multilineage erythroid-myeloid-lymphoid potential. Thus, *CDX4+*CD1d+ expression within early mesoderm demarcates an early progenitor of HE. These insights may be used for further study of human hematopoietic development and improve hematopoietic differentiation conditions for regenerative medicine applications.

## Highlights

• scRNAseq of early hPSC differentiation reveals a *CDX1/2/4*^+^ *CD1d +* mesodermal population.
• KDR + CD1d + mesoderm efficiently gives rise to hemogenic endothelium with erythroid, myeloid, and lymphoid potential.
• CD1d-derived CD34 + cells robustly express *HOXA7/9.*

## 1 Introduction

A long-held goal of regenerative medicine has been the *in vitro* production of hematopoietic stem cells (HSCs) that can be used therapeutically and as a platform for the study of hematological disease. Despite recent advances in the field of directed differentiation of human pluripotent stem cells (hPSCs), this goal remains unrealized in the absence of transgene expression (Sugimura et al., 2017). This is due, in part, to our limited understanding of the overall complexity and heterogeneity of embryonic hematopoietic development, where multiple, spatiotemporally separated programs have been identified (Ditadi et al., 2017). Broadly, these can be separated into extra-embryonic, erythro-myeloid programs and the intra-embryonic (definitive) multilineage programs, the latter of which gives rise to the HSC from hemogenic endothelium (HE) within the dorsal aorta (Gritz and Hirschi, 2016). However, the mesodermal origin(s) of HE remains poorly defined.

The hPSC differentiation system has identified several markers of early mesoderm with hemogenic potential, such as *KDR* (Kennedy et al., 2007)*, PDGFRA* (Davis et al., 2008); and *APLNR* (Yu et al., 2012). However, these markers do not discriminate between other mesodermal lineages, nor do they define progenitors of the extra-embryonic-like and intra-embryonic-like hematopoietic programs. We have previously identified that mesodermal expression of *GYPA/GYPB* (CD235a/b) demarcates a population within hPSC differentiation cultures that harbors exclusively extra-embryonic-like hematopoietic potential, and that the expression of CD235a/b on this nascent mesoderm is regulated by stage-specific WNT and ACTIVIN/NODAL signaling (Sturgeon et al., 2014). However, the identification of a cell surface antigen that positively identifies a mesodermal population with exclusively definitive hematopoietic potential, but not extra-embryonic-like potential, has remained elusive. WNT-mediated expression of *CDX1/2/4* has been correlated with specification of KDR+CD235a- mesoderm harboring potential for CD34+*HOXA+* intra-embryonic-like cells with hematopoietic potential (Ng et al., 2016, Creamer et al., 2017, Jung et al., 2021). Thus, we aimed to use these *CDX* genes to identify definitive hematopoietic precursors within mesodermal hPSC cultures.

## 2 Materials and methods

### 2.1 hPSC maintenance and differentiations

The hPSC line H1 (WA01; WiCell) was cultured and differentiated as described previously (Sturgeon et al., 2014, Creamer et al., 2017, Kennedy et al., 2012, Ditadi and Sturgeon, 2016). Briefly, hPSCs were MEF-depleted by culturing on Matrigel (BD Biosciences) in hESC media for 24 hr. Embryoid bodies (EBs) were generated by treating hPSCs with trypsin-EDTA (0.05%) for 1 min. Cells were detached by scraping to form small aggregates (6–10 cells). EBs were resuspended in N2/B27-based media (Sturgeon et al., 2012) supplemented with L-glutamine (2 mM), ascorbic acid (1 mM), monothioglycerol (MTG, 4x10^−4^ M; Sigma), holo-transferrin (150 µg/mL), BMP-4 (10 ng/mL). After 24 h, the media was supplemented with bFGF (5 ng/mL) and following 42 h total, the media was changed using the same media above supplemented with CHIR99021 (3 µM) and SB43152 (6 µM) (Ng et al., 2016). Following 72 hrs of differentiation, EBs were disassociated with treatment of 0.25% Trypsin-EDTA (5 min), followed by FACS (BD Biosciences) isolation of KDR + CD235a-CD1d-/+ mesoderm. FACS isolated cells were re-aggregated at 2.5x10^5^() cells/ml and placed in StemPro-34 media (Gibco) supplemented with L-glutamine (2 mM), ascorbic acid (1 mM), monothioglycerol (MTG, 4 × 10^−4^ M), holo-transferrin (150 μg/mL), VEGF (15 ng/mL), and bFGF (5 ng/mL). After 3 days, cultures were fed with an equal volume of StemPro-34 media supplemented as previously described, but with additional IL-6 (10 ng/mL), IGF-1 (25 ng/mL), IL-11 (5 ng/mL), SCF (50 ng/mL), and EPO (2 U/mL), and cultured for an additional 2 days.

### 2.2 Erythro-myeloid potential

Resultant CD34+CD43- cells were isolated by FACS and then assessed for hemogenic potential as described (Ditadi et al., 2015). Briefly, cells were aggregated overnight at a density of 2 × 10^5^() cells/mL in 96 well plates with StemPro-34 media, containing L-glutamine (2 mM), ascorbic acid (1 mM), monothioglycerol (MTG, 4 × 10^−4^ M), holo-transferrin (150 μg/mL), TPO (30 ng/mL), IL-3 (30 ng/mL), SCF (100 ng/mL), IL-6 (10 ng/mL), IL-11 (5 ng/mL), IGF-1 (25 ng/mL), EPO (2 U/mL), VEGF (5 ng/mL), bFGF (5 ng/mL), BMP4 (10 ng/mL), Flt-3L (10 ng/mL), and SHH (20 ng/mL). Aggregates were then transferred onto Matrigel-coated plasticware cultured for an additional 8 days in the same media. All differentiation cultures were maintained in a 5% CO_2_ and 5% O_2_ environment. All recombinant factors are human and were purchased from R&D Systems (Minneapolis, MN). Analysis of hematopoietic colony forming cell potential via Methocult (StemCellTechnologies) was performed as described previously (Ditadi et al., 2015).

### 2.3 T-lymphoid assay

T-lymphoid potential was assessed using OP9-DLL4 stroma (La Motte-Mohs et al., 2005, Schmitt et al., 2004) as described previously (Sturgeon et al., 2014, Kennedy et al., 2012, Ditadi et al., 2015). Briefly, 1 × 10^4^ CD34+CD43- cells were added to individual wells of a 24-well plate containing OP9-DLL4 cells and cultured alpha-MEM supplemented with 20% FBS, SCF (30 ng/mL), FLT3L (5 ng/mL) and IL-7 (5 ng/mL). After 5 days, cultures were maintained in 6-well plates of OP9-DL4 in the absence of SCF. Every four to five days co-cultures were transferred onto fresh OP9-DL4 cells by vigorous pipetting and passaging through a 40 µm cell strainer. Cultures were assayed following 28 days for the presence of a CD45+CD56-CD4+CD8+ population. Cultures exhibiting at least 100 CD4+CD8+ cells were considered positive for T-lymphoid potential.

### 2.4 Single cell RNAseq analyses

Day 3 differentiation cultures were dissociated and immediately fixed with methanol as previously described (Alles et al., 2017). Briefly, EB’s were treated with trypsin-EDTA for 5 min, stopped with 5% FBS + IMDM, and spun at 300 × g for 5 min. After resuspending with PBS + 5% FBS and counting, cells were pelleted at 300 × g for 5 min at 4 °C, the supernatant was removed manually, and the cell pellet resuspended in 2 volumes (200 μl) of ice-cold PBS. To avoid cell clumping, 8 volumes (800 μl) of methanol (grade p.a.; pre-chilled to –20 °C) were added dropwise, while gently vortexing the cell suspension (final concentration: 80% methanol in PBS). The methanol-fixed cells were kept on ice for a minimum of 15 min and then stored at –80 °C. For rehydration, cells were kept on ice, pelleted at 1000 × g, washed and resuspended in PBS + 0.01% BSA, passed through a 40-μm cell strainer, counted and diluted for library prep (1000 cells per µL). Libraries were prepared following the manufacturer’s instruction using the 10X Genomics Chromium Single Cell 3′ Library and Gel Bead Kit v2 (PN120237), Chromium Single Cell 3′ Chip kit v2 (PN-120236), and Chromium i7 Multiplex Kit (PN-120262). 17,000 cells were loaded into a well of the chip, capturing > 6000 cells. cDNA libraries were sequenced on an Illumina HiSeq 3000. Sequencing reads were processed using the Cell Ranger software pipeline (version 2.1.0). Using Seurat (Stuart et al., 2019) (version 3.9.9) implemented in R (version 4.0.3) for all steps described below, the dataset was filtered by removing genes expressed in <3 cells, and retaining cells with unique gene counts between 200 and 6000. The remaining UMI counts were normalized and transformed, and regression was performed to account for the percent of mitochondrial UMI counts. Principal component analysis was used to generate uniform manifold approximation and project (UMAP) plots and unsupervised clustering was performed using a resolution of 0.9, resulting in 12 cell clusters. One cluster was removed for excess mitochondrial gene contribution (median > 10%). Differential gene expression analysis was performed using the ‘FindAllMarkers’ function with a minimum expression threshold of 25%, 0.25 log2 fold change, and p_adj_ (Benjamini-Hochberg) < 0.01 to determine the cell identity of each cluster. The dataset is publicly available at the Gene Expression Omibus (GEO) under the accession number: **GSE139850**.

### 2.5 RNA expression analysis

For qRT-PCR, total RNA was isolated with the RNAqueous RNA Isolation Kit (Ambion), followed immediately by reverse transcription into cDNA using random hexamers and Oligo (dT) with Superscript III Reverse Transcriptase (Invitrogen). Real-time quantitative PCR was performed on a StepOnePlus thermocycle (Applied Biosystems), using Power Green SYBR mix (Invitrogen). Gene expression values were calculated using the ΔC_T_ method against the endogenous control (*ACTB*). Primer sequences are *ACTB* F: AAACTGGAACGGTGAAGGTGACAG R: CAATGTGCAATCAAAGTCCTCGGC. *CDX1* F: TGAACGGCAGGTGAAGATCT R: CTTGTTCACTTTGCGCTCCT. *CDX2* F: GCAAGGTTTACACTGCGGAA R: GGGTTCTGCAGTCTTTGGTC. *CDX4* F: CCTTTCCGAGAGACAGGTGA R: CACCGAGCCTCCACTATTCT. *HOXA7* F: AGGACTGTGGAGATGCTTCC R: AGGAAACATCAGGGCGTACA. *HOXA9* F: CGAGAGGCAGGTCAAGATCT R: TGGCATCACTCGTCTTTTGC. *RUNX1* F: CGTGCACATACATTAGTAGCACTACCTTTG R: CCTCCACGAATCTTGCTTGCAGAGGTTAAG.

### 2.6 Flow cytometry and cell sorting

Antibodies used include KDR-PE/KDR-PE-Cy7 (clone 89106, R&D systems), CD4-PerCP-Cy5.5 (clone RPA-T4), CD8-PE (clone RPA-T8), CD34-APC (clone 8G12), CD34- PE-Cy7 (clone 4H11), CD43-PE (clone 1G10), CD45-APC-Cy7 (clone 2D1), CD56-APC (clone B159), CD235a-APC/PE (clone HIR-2), and CD1d-APC (Clone CD1d42). All antibodies were purchased from BD Biosciences (San Diego, CA) unless stated otherwise. Cells were sorted with a FACSAria™II (BD) cell sorter and analyzed on a Fortessa (BD) cytometer.

## 3 Results and discussion

**Fig. 1:**
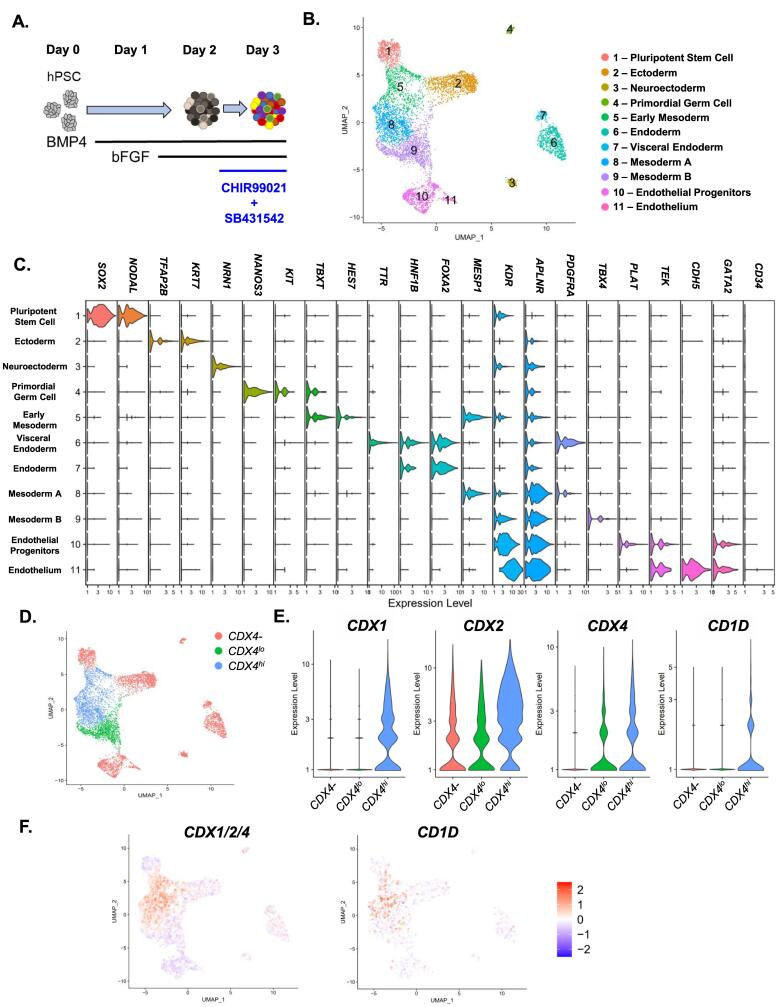
**scRNAseq allows for characterization of *CDX +* mesoderm and reveals *CD1D* as a potential surface marker: A.** hPSC’s were differentiated following exposure to stage-specific BMP4, bFGF, CHIR99012, and SB43152 treatment. On day 3, the differentiation culture was harvested for scRNAseq. **B.** Seurat v3 R package was used to organize the cells into high dimensional space via UMAP and algorithmically distinct clusters denoted by different colors labeled with a putative cell type. **C.** Expression of differential lineage markers in violin plots broken down by cluster, scale = log10 (transcripts). **D.** UMAP of combined clusters with *CDX4-, CDX4^lo^, CDX4^hi^* groups as determined by global DEG analysis. **E.** Violin plots of the expression of *CDX* genes and *CD1D* within different groups, scale = log2 (transcripts). **F.** Expression of *CDX1/2/4* and *CD1D* genes using the module function in Seurat v3 shown on the UMAP where red indicates higher expression and blue lower than expected by chance by random sampling of 5 genes. (For interpretation of the references to colour in this figure legend, the reader is referred to the web version of this article.)

As we previously identified a critical role for mesodermal *CDX4* expression during the hematopoietic differentiation of hPSCs (Creamer et al., 2017), we first sought to characterize the expression of *CDX1/2/4* at a single cell-level within hPSC-derived mesoderm under WNT-dependent intra-embryonic-like culture conditions. To facilitate a better understanding of the overall heterogeneity as well as *CDX* expressing cells within nascent hPSC-derived mesoderm, we leveraged single cell (sc)RNAseq. We assayed cultures on day 3 of differentiation, which contain KDR + CD235a- mesoderm that expresses *CDX* genes in a WNT-dependent manner (Creamer et al., 2017) (Fig. 1**A**). These cultures are at a distinct developmental stage that precedes hemato-endothelial specification (Sturgeon et al., 2014, Ng et al., 2016, Kitajima et al., 2016), which we refer to as “hemogenic mesoderm”. Following standard data processing and UMAP generation, unsupervised clustering was performed using Seurat (Stuart et al., 2019) which identified 11 distinct clusters (Fig. 1**B**). To resolve the identity of many of these clusters, we identified differentially enriched genes (DEGs) within each cluster, revealing a striking degree of heterogeneity within the cultures (**Supplementary Table 1**). Distinct transcriptional identities for many developmental intermediates could be identified in each cluster, including residual pluripotent stem cells (*SOX2, NODAL*), ectoderm (*TFAP2B, KRT7, NRN1*), neuroectoderm (*NRN1*), primordial germ cell (*NANOS3, KIT*), early mesoderm (*TBXT, HES7),* endoderm (*HNF1B, FOXA2*), visceral endoderm (*TTR*), mesoderm (*MESP1*, TBX4, *OSR1*), and endothelial progenitors / endothelium (*PLAT, TEK, CDH5, GATA2*) (Fig. 1**C**). As the “Early Mesoderm”, “Mesoderm A” and “Mesoderm B” (clusters 5–9 respectively) all expressed markers associated with early mesoderm (*KDR, APLNR, PDGFRA*), but preceded hemato-endothelial specification (*TEK, CDH5, GATA2*; Fig. 1**C**) (Sturgeon et al., 2014, Creamer et al., 2017), we focused on these cells as candidate hemogenic mesodermal populations.

We and others (Ng et al., 2016, Creamer et al., 2017, Jung et al., 2021, Davidson et al., 2003, Wang et al., 2008) have previously demonstrated that early expression of *CDX1/2/4* correlates with definitive hematopoietic specification, and that *CDX4* acts as critical regulator of HE development from its mesodermal precursor (Creamer et al., 2017). We therefore hypothesized that *CDX(4)*+ clusters may contain these precursors to definitive HE, and could be used to identify unique cell surface antigens for their identification *in vitro*. Differential gene expression testing performed on each cluster compared to all remaining clusters revealed that *CDX4* was positively enriched only within the “Early Mesoderm” (cluster 5) and “Mesoderm A” (cluster 8), but not within “Mesoderm B” (**Supplementary Table 1**). This led us to designate “Early Mesoderm” and “Mesoderm A” as the “*CDX4^hi^”* clusters within the dataset. While “Mesoderm B” did not have significantly enriched *CDX4* in comparison to the rest of the dataset, low level *CDX4* expression was detectable, leading us to designate this cluster as “*CDX4^lo^”,* and the remaining clusters, comprised of all other cell types, as “*CDX4-“* (Fig. 1**D/E**). Interestingly, when differential gene analysis was performed across these newly defined groups, *CD1D*, a non-canonical MHC receptor found on antigen presenting cells (Beckman, 1994), was found to be enriched in the *CDX4^hi^* clusters and correlated with high *CDX1/2/4* expression (**Supplementary Table 2,** Fig. 1**F**).

**Fig. 2:**
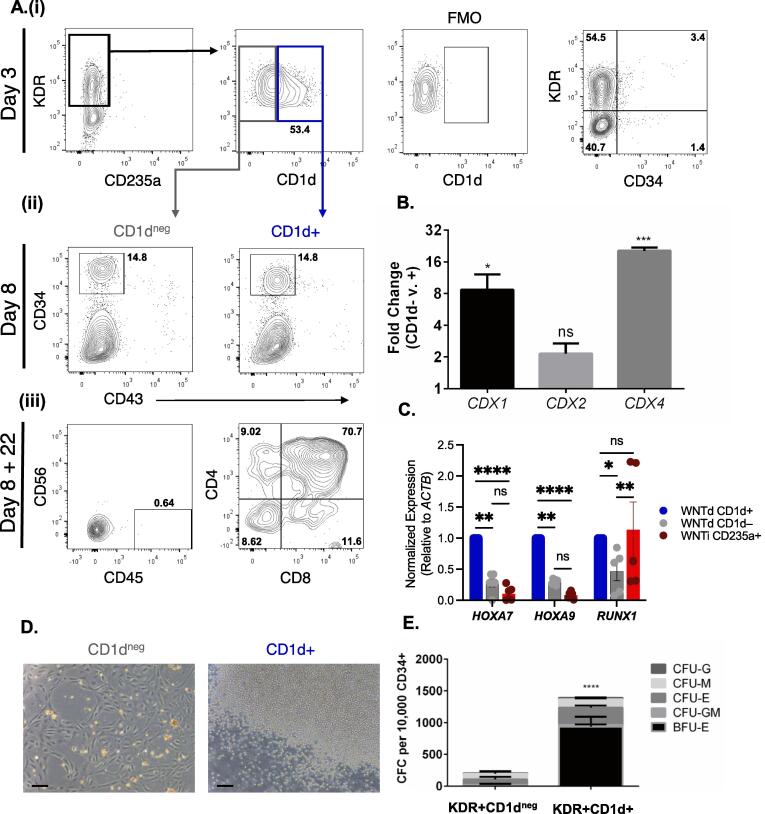
**Phenotypic and functional characterization of CD1d expressing cells within day 3 mesoderm A.(i)** Representative flow cytometry of day 3 differentiation cultures for KDR, CD235a, CD34, and CD1d. KDR+CD235a- definitive hemogenic mesoderm was then assessed for the expression of CD1d (*n* = 6). **(ii)** Representative flow cytometry plots of cultures as in **(i)**, reaggregated for an additional 5 days (*n* = 6). Each resultant culture was assessed for CD34 and CD43 expression. CD34+CD43- cells were isolated by FACS. **(iii)** Representative flow cytometry assay for T cell potential of each culture as in **(ii)**. Following 22 days of coculture, cells were harvested for flow cytometric analysis of CD45+CD56- population for CD4 and CD8 expression, *n* = 3. **B.** Fold change in expression of *CDX* genes via qPCR in KDR+CD235a-CD1d^neg^/+ FACS isolated cells on day 3 of differentiation. *n* = 3, +/- SEM, * p < 0.05, *** p < 0.001 via students *t* test. **C.** Normalized expression levels of *HOXA7, HOXA9, and RUNX1* genes, as determined by qRT-PCR, within CD34+CD43- cells derived from KDR+CD1d^neg^/+ or KDR+CD235a+ mesoderm. *n* = 6 (n = 5 for *RUNX1*), +/- SEM, * p < 0.05, ** p < 0.01, and **** p < 0.0001 via Turkey’s Multiple comparisons test. **D.** Representative micrographs of cultures of isolated CD34+ CD43- cells, as in **Aii**, cultured as a monolayer for another 9 days in endothelial to hematopoietic transition (EHT) promoting media, 100X magnification scale bar = 100 µm. **E.** Erythro-myeloid potential of EHT cultures as in **C.** Cultures were harvested and placed into hematopoietic methylcellulose media for colony forming assays. The numbers of colonies were then assessed after 10–12 days and the numbers were counted of burst forming units erythroid (BFU-E) and colony forming units (CFU) of erythroid (E), granulocyte (G), myeloid (M), and mixed granulocyte/myeloid (GM). *n* = 6, +/- SEM, **** p < 0.0001 2-way ANOVA.

Flow cytometric analysis of these differentiation cultures on day 3 following CHIR99021 and SB431542 treatment, 61.47 +/- 3.121% (SEM, *n* = 6) of KDR + CD34-CD235a- mesoderm was CD1d+ (Fig. 2**A)**. While FACS-isolated CD1d + cells were 8-fold enriched and 20-fold enriched respectively in *CDX1* and *CDX4* expression (Fig. 2**B**), there was a non-statistically significant ∼2-fold enrichment of *CDX2 (p = 0.0545)*. This agrees with the observation in the scRNAseq dataset that the *CDX4-* and *CDX4^lo^* populations also expressed *CDX2* but lacked any *CD1D* expression (Fig. 1**E/F**). To determine the hematopoietic potential of each mesodermal population, KDR+CD235a-CD1d^neg^/+ cells were isolated by FACS and cultured an additional 5 days under hemato-endothelial promoting conditions (Sturgeon et al., 2014). Interestingly, each population gave rise to a CD34+CD43- population at similar frequencies (Fig. 2**Aii**). Gene expression analysis of isolated CD34+ endothelium from each culture revealed that the CD1d+ derived endothelium was significantly enriched for *HOXA7/9* and *RUNX1* expression in comparison to those derived from CD1d^neg^ mesoderm (Fig. 2**C**), suggesting that hemogenic potential may be restricted to the CD1d-derived CD34+ cells. We therefore isolated each by FACS to assess for the presence of functional HE (Ditadi et al., 2015). After culturing as a monolayer for an additional 9 days under endothelial-to-hematopoietic transition (EHT) promoting conditions, the CD1d^neg^-derived CD34+ cells exhibited no signs of multilineage hematopoietic potential. However, the CD1d+ -derived CD34+ cells exhibited robust hematopoietic potential, with many round non-adherent hematopoietic cells observed in the culture (Fig. 2**D**). These cells were confirmed to be hematopoietic progenitors, as when we transferred this population to methylcellulose, many erythro-myeloid CFU were detected (Fig. 2**E**).

Having observed that nearly all definitive *erythro*-myeloid lineage potential was contained within the KDR+CD235a-CD1d+ mesoderm, we next wanted to assess the presence of T-lymphoid potential, as multilineage erythro-myeloid and T-lymphoid potential are a defining characteristic of hPSC-derived definitive hematopoiesis (Kennedy et al., 2012). After isolation of KDR+CD235a-CD1d^neg^/+ cells on day 3, CD34+CD43- progenitors were further FACS purified on day 8 and co-cultured with OP9-DL4 stroma (La Motte-Mohs et al., 2005, Schmitt et al., 2004) under T-cell promoting conditions (Sturgeon et al., 2014, Kennedy et al., 2012, Ditadi et al., 2015). After 22 days, flow cytometric analysis confirmed that only CD34+ cells derived from CD1d+ mesoderm were able to give rise to CD45+CD56-CD4+CD8+ T-cells *in vitro* (Fig. 2**Aiii**). Collectively, these results demonstrate that CD1d demarcates nascent hemogenic mesoderm with definitive hematopoietic potential.

In summary, single cell transcriptomics of early stage hPSC differentiation cultures has revealed a subpopulation of mesoderm, preceding hemato-endothelial specification, that is highly enriched for both *CDX4* and *CD1D*. Subsequent functional *in vitro* studies demonstrate that CD1d is a marker for *CDX4^hi^* hemogenic mesoderm with multilineage definitive erythroid, myeloid, and lymphoid potential. While this does not demonstrate a cell-autonomous role for *CDX4* in the development of the definitive hematopoietic program, the strong correlation between CD1d and *CDX4* expression, coupled with our previous studies demonstrating that *CDX4* regulates definitive hematopoietic development within mesoderm, is consistent with the hypothesis that CD1d identifies *CDX4+* hemogenic mesoderm. Notably, not all CD1d+ cells acquire CD34 expression and develop into HE (Fig. 2**A**), suggesting that either there is significant unappreciated heterogeneity within the CD1d+*CDX4*+ mesoderm, or that the development of non-hematopoietic lineages from a common precursor, similar to the hemangioblast (Choi et al., 1998), which possibly serves a supportive role for definitive hematopoietic development.

It also remains to be determined which mesodermal lineage(s) arise from CD1d^neg^ mesoderm. It will be of great interest to determine if there are functional differences in the CD34+ endothelial progeny from CD1d+ and CD1d^neg^ mesoderm. For example, our scRNAseq analyses show that the *CDX4*^lo^ cluster is enriched in cardiogenic genes such as *TBX2/4* and *HAND1/2* (**Supplementary Table 2**). One possibility is that CD1d^neg^ mesoderm harbors endocardial potential, which is consistent with observations that early *CDX(1/4)* expression is negatively correlated with cardiogenic potential *in vivo* and *in vitro* (Lengerke et al., 2011).

Finally, the *CDX4*^hi^ mesodermal population we identified exhibits enrichment for *HOXA1* and *HOXA3*, of which the latter is a regulator of HE development (Iacovino et al., 2011). Curiously, the *CDX4^lo^* population was enriched for *HOXA9, HOXA10, HOXA11,* and *HOXA13,* suggesting there are differences in distal/proximal *HOX* gene expression within each mesodermal subset (**Supplementary Table 2**). *HOXA* cluster expression has also been proposed as an important gene element of definitive HE *in vitro* and *in vivo* (Ng et al., 2016, Jung et al., 2021), but it is unclear which elements are necessary and/or sufficient for the emergence of HSCs. ‘Medial’ *HOXA* genes (*HOXA5/7/9*) have been demonstrated to be important for nascent HSCs (Dou et al., 2016) and, interestingly, both *CDX4^hi^* and *CDX4*^lo^ populations are enriched for *HOXA7*. Distinct states of *HOXA* gene patterning were also observed when CD1d^neg^/+ mesoderm was isolated and differentiated into CD34+ endothelium. This evidence reinforces the critical role of gene specification early in differentiation and that appropriate patterning of medial *HOXA* expression within hemogenic mesoderm via additional signal pathway manipulation may ultimately prove critical in the derivation of HSCs from hPSCs.

We previously demonstrated that expression of both KDR and CD235a, prior to CD34 acquisition, identifies a mesodermal population that harbors exclusively extra-embryonic-like hematopoietic potential (Sturgeon et al., 2014). Here, we show that the combined markers of KDR, CD235a, and CD1d can identify a hemogenic mesodermal population with exclusively definitive hematopoietic potential. With this insight, mid- and high-throughput screening techniques can be applied to identify novel signal pathways that regulate definitive hematopoietic development, which in turn, should enable the development of technologies for the *in vitro* specification of HSCs from hPSCs.

## Declaration of Competing Interest

The authors declare the following financial interests/personal relationships which may be considered as potential competing interests: Christopher Sturgeon reports a relationship with Clade Therapeutics, Inc. that includes: board membership.

## Data Availability

Data will be made available on request.
